# Intergenerational Engagement in Residential Settings: A Scoping Review of the Literature

**DOI:** 10.1093/geroni/igab046.3090

**Published:** 2021-12-17

**Authors:** Andrea Gardiola, Raza Mirza, Amanda Bull, Christopher Klinger, Jessica Hsieh, Samantha Peck

**Affiliations:** 1 Queen's University, Toronto, Ontario, Canada; 2 University of Toronto, Toronto, Ontario, Canada; 3 University of Toronto, Whitby, Ontario, Canada; 4 Family Councils of Ontario, Toronto, Ontario, Canada

## Abstract

Intergenerational engagement provides a rich environment for people of different ages to come together and exchange life stories, skills, and knowledge. Today, intergenerational interactions are decreasing, however, these exchanges can have positive implications for seniors in residential care homes (RCHs) and younger persons. A scoping review following Arksey and O’Malley’s five-step framework was conducted to investigate the impact of intergenerational engagement and programs (IGPs) on older adults in RCHs. A systematic search of ten electronic databases and hand search of references was carried out; thematic content analysis to established key themes. A total of 1,183 academic and grey literature sources were reviewed, with 66 full-text studies assessed for eligibility. Of these sources, 35 studies met inclusion criteria. Studies highlighted four main themes: 1. Types of IGPs, 2. Psycho-social benefits for older adults and improved status among elders with cognitive impairments, 3. Younger person benefits, suggesting reduced ageism and improved social and communication skills, and 4. Program recommendations, including the need for enthusiastic program facilitators, coordination between facilities, sensitivity training for younger persons, detailed advertisements, and appropriate activities for different age groups. Findings inform future practice and research, highlighting that IGPs are an effective strategy to alleviate negative health outcomes for seniors in RCHs. Future research is needed to evaluate long-term effects and further health outcomes. IGPs provide an opportunity to facilitate purposeful and reciprocal relationships between generations, fostering intergenerational understanding. By studying IGPs and intergenerational interactions, we can better determine practices that meaningfully engage elders in RCHs in Canada.

